# Engineering *Is*PETase and Its Homologues: Advances in Enzyme Discovery and Host Optimisation

**DOI:** 10.3390/ijms26146797

**Published:** 2025-07-16

**Authors:** Tolu Sunday Ogunlusi, Sylvester Sapele Ikoyo, Mohammad Dadashipour, Hong Gao

**Affiliations:** 1 School of Health and Life Sciences, Teesside University, Middlesbrough TS1 3BA, UK; ogunlusitolu@gmail.com (T.S.O.); sylvosly@gmail.com (S.S.I.); 2 National Horizons Centre, Teesside University, Darlington DL1 1HG, UK

**Keywords:** polyethylene terephthalate, PETase, heterologous expression, in silico screening, enzyme assays, host optimisation

## Abstract

Polyethylene terephthalate (PET) pollution represents a significant environmental challenge due to its widespread use and recalcitrant nature. PET-degrading enzymes, particularly *Ideonella sakaiensis* PETases (*Is*PETase), have emerged as promising biocatalysts for mitigating this problem. This review provides a comprehensive overview of recent advancements in the discovery and heterologous expression of *Is*PETase and closely related enzymes. We highlight innovative approaches, such as in silico and AI-based enzyme screening and advanced screening assays. Strategies to enhance enzyme secretion and solubility, such as using signal peptides, fusion tags, chaperone co-expression, cell surface display systems, and membrane permeability modulation, are critically evaluated. Despite considerable progress, challenges remain in achieving industrial-scale production and application. Future research must focus on integrating cutting-edge molecular biology techniques with host-specific optimisation to achieve sustainable and cost-effective solutions for PET biodegradation and recycling. This review aims to provide a foundation for further exploration and innovation in the field of enzymatic plastic degradation.

## 1. Introduction

One of the greatest innovations of the 20th century, plastics have become indispensable to human existence because of their advantageous qualities. Since 2000, global plastic production has doubled from approximately 200 million tonnes (Mt) to ~400 Mt in 2019 [1]. Unfortunately, because plastics are not biodegradable, plastic trash builds up in landfills and the ocean, which causes major environmental issues [2].

On a global scale, the recycling rate for plastic garbage stands at a modest 18%, with a slightly higher proportion of 24% being submitted to burning. The other 58% of plastic waste is either deposited in landfills or released into the natural environment, resulting in the long-term buildup and persistence of plastics [3]. Plastics can be classified into two distinct groups: biodegradable plastics, which can come from either natural or synthetic sources and possess the ability to decompose through the action of microorganisms, and nondegradable plastics, which are derived from petrochemicals and exhibit a higher molecular weight due to the repetitive arrangement of small monomer units, commonly known as synthetic polymers. Illustrations of the latter classification include polyethylene (PE), polypropylene (PP), polyvinyl chloride (PVC), polystyrene (PS), polyethylene terephthalate (PET), and other plastics [4].

### 1.1. PET

With an annual output of around 50 Mt, PET has become one of the most used synthetic polymers since its invention in the 1940s and has emerged as an important contributor to the growing problem of plastic waste. The primary cause of its propensity for degradation is largely attributed to the existence of hydrolysable ester linkages in its composition [5].

Physical and chemical approaches have been implemented to degrade plastic waste, such as ultraviolet (UV) treatment, physical stress, hydrolysis, and ammonolysis [6]. However, these approaches all have some limitations. For example, physical treatments degrade plastics into smaller fragments, thus will not significantly reduce the plastic entering the landfill flow, and the additives in the plastic will also be degraded to release substances that may affect the environment [7]. Chemical treatment requires the use of solvents that are not environmentally friendly and always result in the release of byproducts such as carbon monoxide (CO), carbon dioxide (CO_2_), terephthalic acid, anhydrides, carboxylic acids, and esters [8].

To confront the imminent peril of plastic accumulation in marine and terrestrial environments, it is crucial to implement efficient biodegradative methods that alleviate the burden of plastic in the environment.

### 1.2. PETase from Ideonella sakaiensis

PET does not biodegrade well and instead accumulates as garbage. It was recently discovered that bacteria and fungi possess thermophilic hydrolases, which are best suited for catalysing PET hydrolysis at elevated temperatures. Remarkably, enzymes derived from *Ideonella sakaiensis* were reported to digest PET well at room temperature [9].

PETase, derived from *I. sakaiensis*, also identified as *Is*PETase, is an enzyme that has similarities to cutinase. It has attracted attention as a potentially effective and innovative approach to completely break down the polymer into its fundamental constituent units. *Is*PETase exhibits increased activity at ambient temperatures and acts specifically on highly crystalline PET [10], while the high-resolution (0.92 Å) X-Ray crystal structure of *Is*PETase provided more information on the enzyme’s working mechanism and suggested that it is not fully optimised for crystalline PET degradation [11].

There are concerns about the structural and sequence characteristics that enable *Is*PETase to outperform its homologues at lower temperatures, since *Is*PETase can break down PET films at ambient temperature, whereas other hydrolases require a high temperature to promote PET hydrolysis [9]. Although with these advantages, the practical implementation of *Is*PETase in the biodegradation of PET is hindered by its low solubility [12], highlighting the need to screen more similar enzymes to make *Is*PETase a more feasible target to be engineered for PET’s biodegradation.

This review provides a focused synthesis of recent advances in the discovery and engineering of PET-degrading enzymes, particularly homologues of *Is*PETase. We explore innovative in silico and AI-driven screening strategies, evaluate diverse enzymatic activity assays, and critically assess recombinant expression systems across bacterial, yeast, and algal hosts. Emphasis is placed on enhancing enzyme solubility, secretion, and activity through molecular and host-specific engineering approaches. By highlighting both the progress and persistent challenges in PETase development, this review aims to guide future research toward scalable, sustainable solutions for enzymatic PET biodegradation and recycling.

## 2. Screening *Is*PETase Homologues

### 2.1. In Silico Screening

#### 2.1.1. Amino Acid Sequence-Based Screening

**Figure 1:**
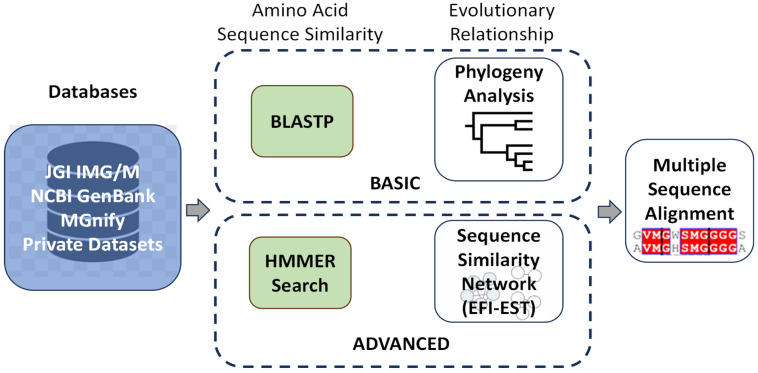
Strategies to search novel *Is*PETase homologues. This schematic illustrates the main computational approaches used to identify potential PET-degrading enzymes from genomic and metagenomic databases. The strategies are broadly categorised into amino acid sequence-based screening, which includes tools such as BLASTP to identify homologous sequences based on similarity to *Is*PETase. Hidden Markov Models (HMMs) are also employed to detect conserved motifs across large datasets.

Several research teams have employed in silico-based methodologies to screen enzymes exhibiting a high level of similarity to *Is*PETase, sourced from a diverse range of microorganisms or environmental samples. The objective of these efforts is to identify potential candidates for further genetic engineering. Researchers hope to accelerate the degradation process of PET [13,14,15]. These screening approaches, as outlined in Figure 1, offer a promising pathway towards the development of more effective biocatalysts for environmental applications in plastic waste management.

Studies employing the strategies outlined in Figure 1 have been published. However, only a tiny fraction of these investigations resulted in confirmed enzymatic activity of the newly identified candidates, leaving further validation still required [14,16,17,18,19].

Many studies have utilised the NCBI (National Center for Biotechnology Information; http://ncbi.nlm.nih.gov) database for enzyme screening, employing a variety of in silico strategies. These range from simpler methods such as BLSTP [15] to more advanced techniques like PSI-BLAST [19,20]. These approaches are particularly appealing to researchers without extensive bioinformatic expertise, as they are relatively straightforward to implement. Remarkably, they have proven effective in identifying promising enzyme candidates, including novel enzymes whose activities have been experimentally validated.

For instance, a more comprehensive strategy was adopted by searching across multiple databases, including NCBI, UniProt, and the JGI Integrated Microbial Genomes and Microbiomes (JGI/IMG) database [14]. The researchers employed an *Is*PETase-specific Hidden Markov Model (HMM)-based search algorithm, which enabled the discovery of novel *Is*PETase homologues. Similarly, the MGnify database, another resource for microbiome data, was explored using an HMM-based approach [17]. Both studies successfully identified enzymes that exhibited activity against PET, highlighting the efficiency of this search technique in uncovering enzymes with plastic-degrading capabilities.

In contrast to the broad database-wide searches, other researchers have focused their efforts on specific groups of microorganisms within databases. For example, the basic BLASTP strategy was applied to 52 Streptomyces genome sequences obtained from NCBI, which included 23 terrestrial and 29 marine isolates. The work targeted approach led to the discovery of an *Is*PETase-like enzyme, whose activity was confirmed through experimental assays [16]. On the other hand, BLAST searches were conducted across 7375 terrestrial metagenomic samples in the JGI/IMG database [13] (https://img.jgi.doe.gov/). While homologues closely related to *Is*PETase were identified, the activity of these candidates was not tested, leaving their potential for PET degradation unverified.

These diverse strategies demonstrate the adaptability and potential of in silico screening in identifying promising enzymes for further study in PET biodegradation research.

#### 2.1.2. Artificial Intelligence (AI)-Driven and Structure-Based Screening

In addition to engineering *Is*PETase, AI tools have also been employed for PETase screening. However, the limited number of experimentally confirmed PETases presents a challenge for effective model training. To address this limitation, existing studies have devised various strategies to enhance training datasets, thereby mitigating the need for additional PETase sequences.

One machine learning (ML) model, employing the support vector machine method, was trained using a dataset of PETase homologues derived from thermal metagenomes. The homologues were collected through in silico screening based on amino acid sequences [21], and the ML-assisted screening resulted in the identification of 74 candidate enzymes. Of these, 51 were successfully expressed in *Escherichia coli*, and 37 exhibited detectable PET hydrolytic activity, including 23 enzymes that had not been previously reported.

In a separate study, a Generative Adversarial Network (GAN) was utilised for data augmentation. By employing a pre-trained deep Evolutionary Scale Language Model (ESM), potential PET-degrading enzymes were identified from a custom-built metagenomic library. Ultimately, 21 novel potential PET-degrading enzymes were identified through structure analysis, though enzymatic activity assays were not conducted to confirm their functionality [22].

To enhance the accuracy of in silico screening strategies, structural features were considered during the screening phase, rather than being assessed solely at the confirmation stage. In one study, novel PETases were identified from the AlphaFold protein structure database. Candidate enzymes were selected based on the presence of key *Is*PETase features, including an aspartic acid residue at positions 43–49 and a histidine residue at positions 71–81, following the Ser-Met-Gly-Gly-Gly motif. These candidates were further refined using the catalytic triad C-α mutual distance criterion, yielding an initial selection of 4594 putative PET hydrolases. Subsequent analysis, including sequence similarity network construction and multiple sequence alignment, narrowed this to a final pool of 107 candidate sequences. Although the most promising candidate, LSPET4, did not surpass *Is*PETase in activity, mutated *Is*PETase based on the structure feature of LSPET4 demonstrated a 9.32-fold increase in *Is*PETase hydrolytic activity at 45 °C [23].

Another study developed a novel PETase discovery pipeline by integrating protein language models with a structural representation tree. The most promising enzyme identified, *Kb*PETase, exhibited twice the activity of *Is*PETase at 30 °C. Notably, when compared with FastPETase, the most active *Is*PETase mutant with the optimal catalytic performance at 50 °C, *Kb*PETase still displayed a catalytic efficiency 1.3 times greater than that of FastPETase [24].

### 2.2. Assays to Screen PETase

**Figure 2:**
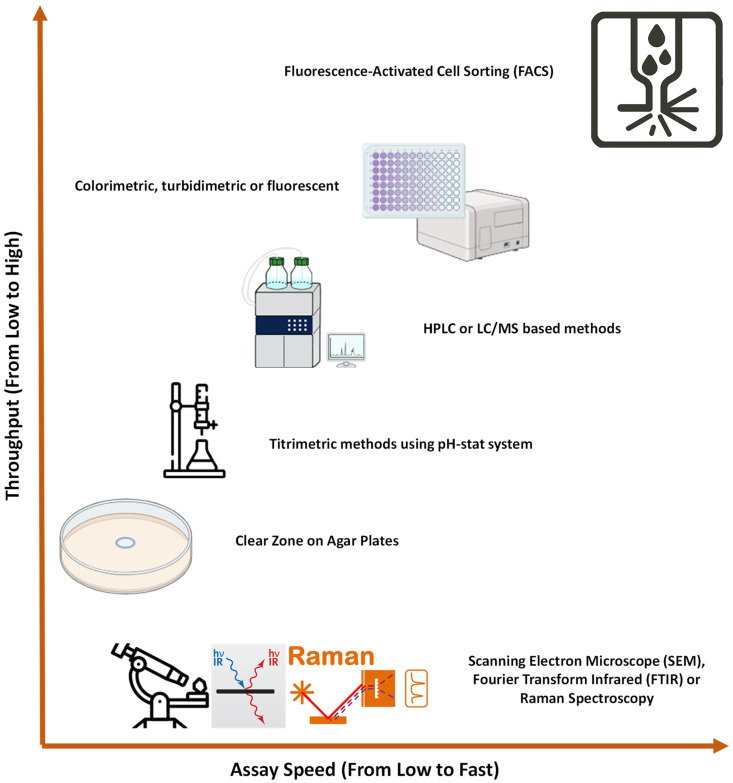
Assays to screen PET-degrading enzymes.

Researchers typically combine in silico approaches with PET-degrading activity assays to screen novel PETases. This dual approach allows them to identify enzymes that are structurally similar to *Is*PETases and capable of degrading PET. The most commonly used assays for measuring PET-degrading activity are depicted in Figure 2. These screening techniques vary in scale from low to ultra-high throughput, depending on the context. High and ultra-high throughput assays are generally employed when screening engineered enzymes resulting from mutation or directed evolution [25,26,27,28,29]. However, when it comes to screening *Is*PETase homologues from environmental samples or databases like NCBI, medium or even low-throughput assays are often more suitable due to the low number of hits from in silico searching. These lower throughput methods strike a balance between efficiency and affordability, making them a more practical choice for such exploratory studies, where fewer samples may be initially tested [14,30].

Building on this framework, researchers tailor their choice of assay based on the nature of the PETase candidates and the experimental constraints. Agar plate-based methods, for example, provide a straightforward and visual means of assessing polymer degradation but are limited by their low throughput [14,30]. Titrimetric methods, including the use of pH-stat systems, provide a moderate-throughput alternative for quantifying acid release during PET hydrolysis [31,32]. HPLC or LC/MS-based assays offer higher specificity and sensitivity, enabling detailed quantification of degradation products, though they require more time and instrumentation [20,33,34].

For high-throughput applications, colorimetric, turbidimetric, or fluorescent assays are widely used due to their compatibility with microplate formats and automation [25,28,35]. At the ultra-high-throughput level, Fluorescence-Activated Cell Sorting (FACS) allows for rapid screening of vast enzyme libraries, particularly in yeast surface display systems [29].

While not typically used for routine screening, scanning electron microscope (SEM), Fourier Transform Infrared spectroscopy (FTIR) and Raman spectroscopy serve as valuable tools in specialised contexts [36,37,38]. These methods are slower, more costly, and lower in throughput, but they provide unique insights into enzyme–substrate interactions and the degradation of crystalline PET. As such, they are often employed to complement biochemical assays in mechanistic or structural studies.

## 3. Heterologous Expression Platforms to Screen PETases

Beyond screening for novel homologues, *Is*PETase itself has been engineered to enhance its properties. Challenges such as inefficient enzyme secretion and cytoplasmic stress have led to the development of secretion expression and surface display platforms in diverse hosts [39]. These advanced platforms facilitate the rapid screening of enzyme variants, eliminating the need for cell lysis and avoiding the formation of inclusion bodies, thereby streamlining the screening process [12,40,41].

The successful expression of *Is*PETase and its homologues in heterologous systems is critical for both fundamental research and industrial applications. Various microbial hosts, including bacteria, yeasts, and microalgae, have been explored for this purpose, each offering distinct advantages and limitations. For example, *E. coli* is widely used due to its rapid growth and well-established genetic tools, while *Bacillus subtilis* offers efficient secretion capabilities. Yeasts such as *Pichia pastoris* and *Saccharomyces cerevisiae* provide eukaryotic post-translational modifications, and microalgae offer environmentally sustainable platforms for enzyme production. In the following subsections, we examine host-specific strategies that have been developed to enhance PETase expression, solubility, and secretion in these systems.

### 3.1. Bacteria

#### 3.1.1. *E. coli*

Most studies that have expressed *Is*PETase or its homologues in *E. coli* have utilised pET-series vectors and BL21 (DE3) or derived strains, such as BL21 (DE3)-T1R [42] and BL21-Gold (DE3) [26], with IPTG added to induce expression.

As an alternative to the widely used *E. coli* BL21 (DE3), *E. coli* SHuffle T7 was engineered for the intracellular expression of *Is*PETase^Mut^, an active mutant of *Is*PETase. This alternative was chosen due to the propensity of proteins with disulfide linkages to misfold and accumulate as inclusion bodies during overexpression, a phenomenon initially observed with the expression vectors pBAD and pET30a [43,44]. This was corroborated by a separate study in which *Is*PETase was expressed in multiple *E. coli* hosts, with SHuffle T7 Express demonstrating the highest enzymatic activity. However, this advantage was not observed for FastPETase (N233K/R224Q/S121E/D186H/R280A) [45] and Hot-PETase [46], two variants of *Is*PETase [47]. An additional noteworthy finding indicated that the formation of inclusion bodies during the overexpression of *Is*PETase^Mut^ in *E. coli* was primarily attributed to protein aggregation driven by interactions among hydrophobic regions, rather than the presence of disulfide bonds [12]. Consequently, the precise role of SHuffle T7 as an expression host requires further elucidation.

A novel, growth-decoupled *E. coli* strain, enGenes Biotech’s e^x^-press V2, was employed in one study to express the PET-degrading enzyme polyester hydrolyse Leipzig 7 (PHL7) [48]. In this system, induction with arabinose inhibits *E. coli* host cell RNA polymerase, thereby arresting cell growth and reallocating cellular resources to enhance recombinant protein production [49]. PET-degrading activity was detected in the unpurified fermentation supernatant; however, enGenes e^x^-press V2 was not benchmarked against established *E. coli* host strains [48].

##### Enhanced Secretion System

A frequently encountered challenge in expressing *Is*PETase in *E. coli* is its low solubility. Current research has explored various strategies to address this limitation.

The Sec-dependent pathway is associated with post- and co-translational translocation of pre-folded polypeptides across the inner membrane [26,42]. Signal peptides employing the Sec-dependent pathway are frequently utilised, including PelB, PhoA, OmpC, OmpF, OmpA, and MalE [50]. Among these, PelB has found widespread commercial use in pET vector expression systems, such as pET20b, 22b, and 26b [50], making it a convenient starting point for enhancing *Is*PETase secretion.

Employing this approach, *Is*PETase was cloned into pET22b(+), which contains the DNA sequence for PelB, and subsequently generated a library via standard error-prone PCR (epPCR) to induce mutations in PelB. Using an assay to evaluate the ability to degrade 4-*p*NPA (*para*-nitrophenyl acetate), they identified mutants *Is*PETase with significantly enhanced activity. The increased PETase concentration in the supernatant correlated with improved activity, exhibiting at least 1.7-fold enhancement across various assays [26].

In another study, multiple Sec-dependent signal peptides, including MalE, LamB, and SurA, were tested and demonstrated that LamB, a maltoporin involved in maltose transportation, facilitated the production of significantly higher levels of extracellular *Is*PETase in *E. coli* [42].

Further advancements in the heterologous expression of *Is*PETase in *E. coli* were achieved by modifying signal peptides with N-terminal enhancers. These enhancers, derived from the endogenous signal peptide of β-fructofuranosidase (β-FFase) originating from *Arthrobacter arilaitensis* NJEM01, proved effective in achieving extracellular synthesis of *Is*PETase with increased enzymatic activity [51].

An intriguing finding in this area arises from the use of native signal peptides. When an *Is*PETase homologue from *Streptomyces* sp. SM14 was expressed in *E. coli* with its native signal peptide; the enzyme was successfully exported, as confirmed by the polycaprolactone (PCL) plate clearing assay [16]. This result underscores the potential of leveraging native signal peptides for effective extracellular expression and secretion in heterologous systems.

##### Chaperone Co-Expression and Fusion Tags

*Is*PETase^Mut^ was co-expressed with three chaperone systems: GroEL/ES, Dnak/DnaJ/GrpE, and TF. The findings revealed that *Is*PETase^Mut^ co-expressed with GroEL/ES exhibited a 12.5-fold increase in expression levels [12]. These results align with previous studies indicating that co-expression with chaperones enhances protein solubility in *E. coli* [52].

An additional approach to avoiding inclusion body formation in *E. coli* involves fusing highly soluble tags to recombinant proteins, such as maltose-binding protein (MBP), transcription termination/anti-termination factor (NusA), thioredoxin (TrxA), and small ubiquitin-related modifier (SUMO) [53]. Previous studies have demonstrated that NusA offers superior soluble expression of cold-adapted *Vibrio* proteins in *E. coli* compared to other fusion tags [54]. Similarly, substantial improvements in the solubility of *Is*PETase^Mut^ were achieved through co-expression with the chaperone GroEL/ES and the use of fusion expression with NusA [12].

In a separate study, a novel fusion tag comprising carbohydrate-binding module 66 (CBM66) was fused with *Is*PETase. This approach resulted in significantly increased expression levels of soluble *Is*PETase, achieving yields of approximately 370 mg/L [55].

More recently, the two strategies were combined to enhance *Is*PETase production. A vesicle nucleating peptide (VNp) tag-based secretion system was utilised in conjunction with the co-expression of the DnaJ-DnaK chaperone team. This combined approach resulted in a marked improvement in the production of FastPETase, an *Is*PETase mutant with enhanced hydrolytic activity [56].

##### Cell Surface Display (CSD) System

Microbial cell surface display systems enable peptides and proteins to be presented on the surface of microbial cells by fusing them with anchoring motifs [57]. The outer membrane-bound fatty acid transporter (FadL) from *E. coli* was previously demonstrated to be effective for an esterase enzyme display on the surface of *E. coli* [58]. When FadL was co-expressed with the CSD system, *Is*PETase exhibited activity in the tributyrin plate clearing assay and demonstrated enhanced thermostability [40].

Building on the advancements in CSD systems, the PgsA protein from *B. subtilis* was employed as an anchoring protein for the heterologous expression of *Is*PETase in *E. coli* [59]. PgsA is part of the enzyme complex that synthesises poly-γ-glutamic acid (PGA) in *B. subtilis* and has been fused with lipase B from *Candida antarctica* (CalB) to display the enzyme in an active form on the cell surface of *E. coli* [60]. The fused protein was expressed in *E. coli*, and it was confirmed that the active form of the *Is*PETase was present on the cell surface of *E. coli* [59].

##### Increasing Membrane Permeability

Colicin families offer the functional diversity of unique cell-killing mechanisms that allow the simultaneous release of recombinant enzymes from cells. Of over 20 colicins, the lysis gene from Colicin E7 was effectively reported to enhance the secretion of intracellular recombinant enzymes from *E. coli* [61]. An *Is*PETase homologue screened from metagenome analysis, F148, was co-expressed with Colicin E7 in *E. coli* BL21 (DE3). Compared with the control strain only expressing F148, during the same period, nearly 3-fold degradation products could be observed, since Colicin E7 enabled protein release simultaneously by obstructing the outer membrane through phospholipase activation [62].

In another study, a comparable strategy was adopted by fusing *Is*PETase with *Thermobifida fusca* cutinase, an *Is*PETase homologue previously reported to degrade phospholipids and thereby increase cell membrane permeability [63]. This approach successfully enhanced enzyme secretion, providing another example of facilitating secretion through the modulation of cell membrane permeability [64].

#### 3.1.2. *B. subtilis*

*B. subtilis* is widely recognised as a robust biotechnological workhorse for the heterologous expression of enzymes. Its non-pathogenic nature and designation as a GRAS (generally regarded as safe) microorganism render it fully compliant with regulatory requirements for industrial applications [65,66]. Similar strategies to those employed in *E. coli* have been adapted for use in *B. subtilis* to enhance the extracellular expression of *Is*PETases.

##### Enhanced Secretion System

The use of five *Bacillus*-derived signal peptides for expressing *Is*PETase was investigated in *B. subtilis* WB600, a strain derived from *B. subtilis* 168 with six protease genes knocked out [67]. The signal peptides tested were amy from *Bacillus amyloliquefaciens*; Sac and AprE, both from *B. subtilis* 168; apr and BprA, both from *Bacillus licheniformis* WX-02. Among these, the signal peptide amy (SPamy) demonstrated the most significant effect, increasing enzyme activity in the cell culture supernatant nearly four-fold [68].

In contrast to studies on *Is*PETase expression in *E. coli*, which consistently reported that the Sec-dependent secretion pathway enhances *Is*PETase secretion, research on *B. subtilis* has yielded conflicting results. In a separate study, the expression of *Is*PETase in *B. subtilis* 168 was examined using three Sec signal peptides (AmyE, Bpr, and SacB) and two Tat signal peptides (PhoD and YwbN), which direct proteins through the twin-arginine translocation (Tat) pathway. However, none of these signal peptides improved *Is*PETase secretion compared to its native signal peptide [69]. Thusfurther research is required to elucidate the discrepancies observed in the findings of these studies.

##### Chaperone Overexpression

In *B. subtilis* CBS2, the hrcA gene, a negative regulator of the dnaK and groESL operons [70,71], was inactivated. However, the protein titre of *Is*PETase was not improved in the chaperone-overexpressing strain. In contrast, a PET hydrolase from the bacterium HR29 (*Bhr*PETase) exhibited an approximately 20-fold increase in production [72].

### 3.2. Yeast

#### 3.2.1. *S. cerevisiae*

Surface display systems have been adopted to enhance the extracellular concentration of *Is*PETase. The anchoring protein GCW51 was successfully utilised to display *Is*PETase on the surface of yeast [73]. In a more recent study, a novel surface display platform was developed based on *S. cerevisiae* spores to display FastPETase [41].

Unlike the cell wall of vegetative *S. cerevisiae* cells, which consists of a two-layer structure comprising β-glucan and mannan, the spore wall features a four-layer structure composed of mannan, β-glucan, chitosan, and dityrosine. Displaying proteins on the surface of *S. cerevisiae* spores offers distinct advantages over traditional cell surface display, as it eliminates the need for transmembrane transport. Furthermore, the unique structure of the spore wall imparts resistance to environmental stresses, as demonstrated by a study, where the displayed FastPETase exhibited significantly improved thermostability [45].

Yeast surface display systems provide a promising approach to producing *Is*PETase, offering the advantages of bypassing enzyme purification, resulting in substantial time and cost savings, and facilitating the reuse of the biocatalyst.

#### 3.2.2. *Yarrowia lipolytica*

*Y. lipolytica* has been identified as a potential host for the expression of *Is*PETase, owing to its ability to assimilate atypical carbon sources, allowing for the assimilation of degradation products from PET [74]. Following the successful expression of *Is*PETase in *Y. lipolytica*, the same group optimised its expression by testing various pre-treatment methods [75]. However, in comparison to other host systems, research in *Y. lipolytica* has yet to progress towards a deeper understanding and optimisation at the molecular biology level.

#### 3.2.3. *P. pastoris*

The methylotrophic yeast *P. pastoris* is widely recognised as a versatile workhorse for the production of heterologous proteins in industrial applications [76,77]. This expression system has been shown to support efficient secretory expression, and its post-translational glycosylation further enhances expression yields by increasing the resistance of glycoproteins to proteolytic degradation [78,79]. In another study, *Is*PETase was successfully expressed in *P. pastoris*, demonstrating significant improvements in the enzyme’s specific activity and thermostability both for native *Is*PETase and FastPETase [80].

In another study, a chaperone co-expression strategy was employed in *P. pastoris* to enhance the production of the FastPETase variant, FastPETase-212/277. A panel of chaperones was evaluated, including protein disulphide isomerase (PDI), thiol oxidase (Ero1), the bZIP transcription factor HAC1, and proprotein convertase Kex2. Among these, Ero1 was identified as the most effective in boosting expression [81].

### 3.3. Microalgae

Microalgae present a compelling option for the synthesis of recombinant plastic-degrading enzymes and various environmental applications, owing to their ubiquitous presence in aquatic systems, minimal production of endotoxins, and ability to grow without the need for supplemental organic carbon [82]. In a study, the photosynthetic microalga *Phaeodactylum tricornutum* was employed to secrete a modified PETase, PETaseR280A, into the surrounding culture medium for PET degradation. The gene encoding PETaseR280A was co-expressed with the signal peptide of *P. tricornutum* alkaline phosphatase. The secreted enzyme demonstrated PET-degrading activity, confirming successful expression and secretion [83]. However, the application of *P. tricornutum* is constrained by its specific growth requirements, including low temperatures, silica as a nutrient, and high salinity.

To overcome these limitations, *Chlamydomonas reinhardtii*, a GRAS-designated freshwater microalga, was employed in a subsequent study for the expression of wild-type *Is*PETase. *C. reinhardtii* was selected due to its faster growth rate and freshwater habitat [84]. While the enzyme’s activity was confirmed, the authors did not specify whether a signal peptide was employed to assist the secretion, and enzyme activity was assessed using cell lysates.

In another study, *C. reinhardtii* was employed as the host organism, with the original signal peptide retained to target the recombinant protein to the thylakoid lumen. This study differed by integrating the *Is*PETase gene into the plastome at a specific site, restoring a photosynthesis-essential gene. This restoration allowed antibiotic-free selection following gene integration [85].

In summary, these findings underscore the potential of bioengineered microalgae for producing *Is*PETase and homologues, offering promising applications in bioremediation strategies for PET-polluted aquatic environments.

## 4. Microbial Expression Systems for *Is*PETases and Homologues

The exploration of microbial platforms for *Is*PETase expression and the advancement of in silico analyses, screening assays, and novel enzyme discovery strategies underscore the rapid progress in this field.

### 4.1. Prokaryotic Hosts

Microbial hosts, particularly *E. coli* (Table 1), remain the backbone for heterologous expression, offering high yields and a broad toolkit for genetic manipulation. Sec-dependent secretion pathways, fusion tags, and chaperone co-expression strategies have been instrumental in improving the solubility, stability, and extracellular secretion of *Is*PETase. Recent integrative approaches, such as combining vesicle nucleating peptides with chaperone systems, have further advanced production levels. However, limitations such as protein misfolding and inclusion body formation necessitate continued optimisation and host-specific engineering.

CSD systems represent a complementary approach, allowing *Is*PETase localisation on the cell membrane and bypassing purification steps. The use of anchor proteins such as FadL and PgsA in *E. coli* has resulted in enhanced enzyme activity and thermostability. While promising, further investigation into the stability, scalability, and cost-effectiveness of CSD systems is essential to translate these findings into industrial applications.

In *B. subtilis*, signal peptide optimisation, such as the use of amy from *B. amyloliquefaciens*, has demonstrated success in increasing extracellular *Is*PETase production. However, conflicting results regarding Sec- and Tat-dependent pathways underscore the complexity of secretion mechanisms in this host. Comparative molecular studies are needed to elucidate these discrepancies and fully harness *B. subtilis* as an efficient expression host with a secretion system. Only the strategies of enhancing the secretion system with signal peptides and co-expressing chaperones have been explored in *B. subtilis* to date (Table 1). Several fusion tags have been developed to facilitate the secretion of target proteins in *B. subtilis*, including cellulase [86], SUMO3 [87], Spy [88], and lysyl tRNA synthetase [89]. Further studies are anticipated to improve *Is*PETase secretion and extracellular production in this expression host.

As *B. subtilis* spores can be readily engineered to display heterologous antigens or proteins on their surface, the CSD system has been adapted for spore-surface display in *B. subtilis* [90]. Regarding the final strategy, which has been successfully employed in *E. coli*, thereby increasing membrane permeability to enhance protein secretion, there are also successful examples in *B. subtilis*. For instance, it has been demonstrated that the addition of Triton X-100 increased the secretion level of γ-cyclodextrin glycosyltransferase by 21.5%, an effect attributed to enhanced cell membrane permeability [91].

Thus, future research could focus on advancing *B. subtilis* as an expression host for *Is*PETases through the development of fusion tag technology, spore–surface display systems, and strategies for modulating cell membrane permeability.

### 4.2. Eukaryotic Hosts

Eukaryotic hosts, including yeasts and microalgae, offer unique advantages due to their advanced post-translational modification systems and regulatory compliance for industrial applications. *P. pastoris* has shown significant potential, with glycosylation improving enzyme stability and chaperone co-expression strategies enhancing yields [81]. Yeast surface display systems, particularly those employing *S. cerevisiae* spores, have innovatively addressed the challenges of purification and environmental stress resistance [92]. Microalgae, such as *P. tricornutum* and *C. reinhardtii*, have emerged as promising platforms for *Is*PETase production in aquatic environments [93,94]. However, challenges related to scalability, nutrient requirements, and environmental conditions must be addressed to maximise the potential of these hosts. Furthermore, only a limited number of strategies have been tested in these systems to enhance *Is*PETase secretion, leaving considerable potential for the exploration and implementation of alternative approaches.

### 4.3. Expression and Production of IsPETase and Homologues in Scaled-Up Systems

The industrial-scale application of *Is*PETase and homologues hinges on the development of robust, high-yield expression systems capable of producing active enzymes under fermentor conditions. While significant strides have been made in engineering *Is*PETase variants with enhanced activity and thermostability, translating these advances into scalable bioprocesses remains a key challenge.

Among the various microbial hosts explored, *E. coli* remains the most widely used due to its rapid growth, well-characterised genetics, and ease of manipulation. However, *P. pastoris* has demonstrated superior capabilities in protein secretion and post-translational modifications. Notably, co-expression of the chaperone Ero1 in *P. pastoris*, combined with a high-cell-density fed-batch fermentation strategy in a 30-L bioreactor, enabled the production of up to 3.23 g/L of secreted FastPETase [81]. This represents one of the highest reported yields for the expression of *Is*PETase and homologues in a eukaryotic system. Although this system demonstrated excellent productivity, it required complex media and extended cultivation times to achieve optimal results.

In *E. coli*, the highest yield to date was also achieved for FastPETase, using a synergistic strategy that combined co-expression of cognate chaperones (DnaK/DnaJ from *I. sakaiensis*) with fusion of VNp6. This approach enabled the secretion of over 2 g/L FastPETase in a 5 L fed-batch fermenter [56].

Despite these promising results, several limitations persist. One of the most significant challenges is the lack of standardisation in reporting outcomes. Many studies use enzyme activity or PET degradation efficiency as proxies for expression levels, which allows researchers to avoid time-consuming purification procedures [95]. While this approach is practical, it complicates direct comparison across studies. This inconsistency in metrics, combined with variations in substrate type, reaction conditions, and quantification methods, hampers reproducibility and comparative assessment.

## 5. Conclusions

In summary, the combination of advanced expression systems, in silico methods, screening assays, and extracellular production strategies is accelerating the development of *Is*PETase for PET degradation. However, the field still faces challenges, including the scalability of production systems, discrepancies in secretion efficiency across hosts, and the need for standardised screening protocols. Interdisciplinary approaches that integrate synthetic biology, computational modelling, and environmental microbiology hold the key to overcoming these challenges and advancing the application of *Is*PETase for sustainable bioremediation. Additionally, exploring the potential of diverse microbial systems could provide novel insights and enhance the robustness of PET degradation strategies. Continued investment in these areas will be crucial for developing effective and scalable solutions to address plastic pollution.

Furthermore, while laboratory-scale advancements in PETase engineering are promising, their translation to industrial-scale applications presents significant challenges. These include the need for cost-effective and high-yield fermentation systems, robust enzyme stability under process conditions, and integration into existing waste management infrastructures. Although various microbial hosts have been explored for the expression of *Is*PETase and its homologues, including *E. coli*, *B. subtilis*, yeasts, and microalgae, comparative studies remain limited, and each system presents distinct advantages and constraints. *E. coli* is the most widely used host due to its rapid growth and genetic tractability, but issues such as inclusion body formation and limited secretion efficiency persist. *B. subtilis* offers better secretion potential but yields inconsistent results across signal peptide systems. Yeasts like *P. pastoris* provide post-translational modifications and improved enzyme stability, while microalgae offer sustainable platforms but are still in early development stages. Despite these advances, the state-of-the-art in *Is*PETase expression under fermentor conditions is still emerging. Limitations such as low solubility, inefficient folding, and suboptimal secretion continue to hinder large-scale application. Regulatory hurdles such as the approval of genetically modified organisms (GMOs) for environmental use, biosafety concerns, and compliance with international standards also pose barriers to implementation. Addressing these issues will require interdisciplinary collaboration among biotechnologists, process engineers, and policymakers to ensure that PETase-based biodegradation strategies are not only scientifically viable but also scalable, safe, and regulatory-compliant.

## Figures and Tables

**Table 1:** Strategies employed to optimise the extracellular production of *Is*PETases or homologues using microbial expression systems.

Microbe	Bacteria		Yeast		Microalgae
	*E. coli*	*B. subtilis*	*S. cerevisiae*	*P. pastoris*	*P. tricornutum*
Enhancing secretion system	√	√			√
Chaperone co-expression	√	√		√	
Fusion tags	√				
CSD system	√		√		
Increasing membrane permeability	√				

Note: √ indicates that the corresponding strategy has been applied to the microbial host for enhancing *Is*PETase expression or secretion.

## Data Availability

The original contributions presented in this study are included in the article. Further inquiries can be directed to the corresponding author.

## Abbreviations

The following abbreviations are used in this manuscript:

4-*p*NA	*Para*-nitrophenyl acetate
β-FFase	β-fructofuranosidase
AI	Artificial intelligence
BHET	Bis(2-hydroxyethyl) terephthalate
CBM66	Carbohydrate-binding module 66
CO	Carbon monoxide
CO_2_	Carbon dioxide
CSD	Cell surface display
ESM	Evolutionary scale language model
epPCR	Error-prone PCR
FACS	Fluorescence-activated cell sorting
FTIR	Fourier transform infrared spectroscopy
GAN	Generative adversarial network
GMO	Genetically modified organism
GRAS	Generally regarded as safe
HMM	Hidden Markov model
HPLC	High-performance liquid chromatography
JGI/IMG	JGI integrated microbial genomes and microbiomes
LC	Liquid chromatography
ML	Machine learning
Mt	Million tonnes
NCBI	National Center for Biotechnology Information
PCL	Polycaprolactone
PDI	Protein disulphide isomerase
PE	Polyethylene
PET	Polyethylene terephthalate
PGA	poly-γ-glutamic acid
PP	Polypropylene
PS	Polystyrene
PVC	Polyvinyl chloride
SEM	Scanning electron microscope
SUMO	Small ubiquitin-related modifier
Tat	Twin-arginine translocation
UV	Ultraviolet
VNp	Vesicle nucleating peptide
